# The legal and ethical aspects of the right to health of migrants in Switzerland

**DOI:** 10.1186/s40985-016-0027-2

**Published:** 2016-10-06

**Authors:** Géraldine Marks-Sultan, Stefanie Kurt, Didier Leyvraz, Dominique Sprumont

**Affiliations:** 1 Institute of Health Law, University of Neuchâtel, Avenue du 1er-Mars 26, 2000 Neuchâtel, Switzerland; 2 National Center of Competence in Research, The Migration-Mobility Nexus, NCCR on the move, SwissCentre for Migration Law, University of Neuchâtel, Neuchâtel, Switzerland; 3 Swiss Centre for Migration Law, University of Neuchâtel, Neuchâtel, Switzerland; 4 Institute of Health Law, University of Neuchâtel, Switzerland and Swiss School of Public Health, Zurich, Switzerland

**Keywords:** Right to health, Migrants, Swiss legal framework, Asylum seekers, Foreign nationals, Constitutional rights

## Abstract

The right to health of migrant populations, whether they are foreign nationals, foreign workers, tourists, asylum seekers or refugees, is enshrined in international human rights treaties. The effectiveness of the implementation of this fundamental right thus lies in national legal frameworks. In spite of its long humanitarian tradition, Switzerland has a strict migration policy, and while it has established a non-discriminatory legal framework for the protection and promotion of the right to health, its laws and regulations sometimes codify differences in treatment between foreign nationals and Swiss residents based on distinct situations. On the basis of shared responsibilities between the Federal State and the 26 cantons, this article describes the Swiss legal and regulatory approach to the right to health, the ways it is currently implemented and the possible vectors for an improved integration of migrants into the health system.

## Background

As international organizations show growing concerns about the health of migrants [1] fleeing the chaos in Syria, Iraq and Afghanistan and piling up on their way to safer European countries, the 2015–2016 refugee crisis has highlighted the lack of an international legal framework providing for uniform standards of protection for migrant populations. One manifestation of this vacuum is the absence of an internationally accepted definition of the word “migrant” [2]. This lack of consensus is observable through the different scopes of the definitions developed by international organizations. They can go as far as to include all individuals who have resided in a foreign country for more than 1 year irrespective of the causes [3], or on the contrary be restricted to individuals who freely take the decision to move to another country for reason of “personal convenience” and without intervention of an external compelling factor [4], thus excluding asylum seekers awaiting refugee status. This need to distinguish asylum seekers, escaping conflict areas, from economic migrants, has recently been reflected in the media [5]. In the absence of dedicated international standards, migrants’ protection is mostly grounded in the general rules of human rights enshrined in international treaties and applicable to all human beings. As we will see, several of them provide for the fundamental right to health. The protection of the right to health of migrants should therefore be sought at the national level through an analysis of national practices.

According to a study conducted in Switzerland between 2010 and 2012 with the aim of assessing the perception migrants [6] had of their health, it appeared that those who had lived in the country for more than 3 years clearly described their state of health in more negative terms than the Swiss resident population [7]. While 87 % of the Swiss resident population reported being in good or very good health [8], around 26 % of Turks, 19 % of Serbians and 15 % of Kosovars [9] with a residence permit felt they were not in good health [10]. In the specific field of asylum seekers, while 86.2 % of Somalians declared being in good health, only 45.1 % of Sri Lankan reported the same [11]. This study is a good starting point to understand how health is a central challenge throughout the migration process as risk factors exist at all stages [12]. Asylum seekers’ poor health conditions at their arrival [13], the question of the possible forced returns of these people or the economic benefits of having healthy foreign workers are essential elements that need to be taken into account by States. In this field, national laws and regulations reflect the difficulties of the States to balance conflicting national concerns: the will to help migrants and the existence of economic and security considerations. As a rather conservative country in Western Europe, with a long humanitarian tradition, Switzerland, as a Federal State, is a good example of how competing interests influence the design of public policies.

As with other European countries, Switzerland hosts a diverse foreign population. In 2014, the foreign population with residence permits in Switzerland accounted for 23.6 % of the total population. This population includes 68 % foreign workers from the 28 European Union Member States (EU28) or the European Free Trade Association (EFTA) States and 31.7 % third country nationals [14]. It also covers asylum seekers which represent 1.22 % of the foreign population, 53.1 % of which will be granted refugee status [15]. Finally, figures collected in 2013 report that about 76,000 individuals, no matter their country of origin, are living in Switzerland without a residence permit [16]; they are qualified as undocumented migrants by the Swiss doctrine [17]. It is observable that Swiss laws refer to foreign nationals, asylum seekers and refugees but never use the term “migrant.”

The highest attainable standard of health was first recognized as a fundamental right for every human being in the Preamble of the World Health Organization (WHO)’s 1946 Constitution. As a member State of WHO, this international agreement is binding for Switzerland, which should therefore act in a way that protects and promotes conditions that ensure that the population is as healthy as possible. While this right was further recognized in other international and regional human rights treaties [18], its enshrinement in article 12 of the 1966 International Covenant on Economic, Social and Cultural Rights, which was ratified by Switzerland on the 18th of June 1992, gave rise to a large legal doctrine [19].

The right to health is neither a right to be in good health [20] nor a mere right to health care. It rather consists of a twofold obligation for States. The first one is a best-effort obligation for each State to establish ethically and culturally acceptable health policies that address current local sanitary needs and plan for measures and resources to promote national health in accordance with its capacities. These policies should, on the one hand, describe the health protective and preventive measures the State takes to promote the underlying preconditions for health [21] and, on the other hand, plan for the best possible functioning of a structured set of quality healthcare institutions that contribute to health recovery [22]. Access to these healthcare institutions, as well as the benefits of the health protective and preventive measures, should be granted to all people without discrimination [23]. Beyond the duty of the States to act in favour of health promotion, the right to health also contains freedoms for individuals that States must protect. They notably include the principle of informed consent for all medical treatments as well as the right to privacy and confidentiality concerning health-related information.

The implementation of the right to health in Switzerland needs to be considered in the specific context of a Federal State, in which regions, the 26 cantons, undertake the largest share of responsibilities in regard to health prevention and promotion as well as the provision of health care to the population. While cantons adopt implementing regulations in areas in which the Federal State has already adopted laws, they have the power to adopt their own health policies, laws and regulations within their scope of authority. This is particularly the case in the field of immigration, where cantons are responsible for granting residence permits in accordance with federal legislation—with the exception of Swiss asylum policy, which is within the authority of the Federal State.

In this article, we will focus on Switzerland’s legal framework for the protection and promotion of the right to health of people living within its territory, and we will observe the extent to which this framework takes into consideration the specific vulnerabilities of asylum seekers and other foreigner nationals that have left their countries to settle in Switzerland for economic reasons.

This article is divided in two parts, the first one goes over the federal legal framework to respect, protect and promote the right to health. The second part will review how cantons exercise their authority.

### The implementation of the right to health at the federal level

The Swiss Constitution is the most important legal document at the federal level. It describes the functioning of the State and its institutions, providing authority to the Federal State and the cantons while imposing limits on these powers and enshrining the fundamental rights of the population. The Swiss Constitution presents the values of the State and ensures the cohesion of the population. In this section, we first present how the Constitution recognizes the right to health (1). Then, as a result of the powers thus bestowed to the Federal State, we analyse the type of measures it adopts to protect and promote the right to health (2).

#### The right to health in the Swiss Constitution

The right to health is enshrined in the 1999 Swiss Federal Constitution through multiple articles. As in most countries in Europe, the right to health in Switzerland is seen as a duty for the State: to take measures to protect and promote the health of its population (articles 41 and 118); to respect and guarantee the exercise of the freedoms associated with medical and research ethics (articles 118b and 119) and a right to assistance in situation of distress (article 12) [24]. Discrimination on the basis of origin, race, sex, age, language, social situation, way of life, religious, philosophical or political believes and psychological or mental deficiencies are prohibited through article 8 of the Constitution. While there still may be differences in the way this right is implemented in different situations, the Swiss Federal Court already recognized in 1925 that there should be no discrimination between Swiss residents and foreigners (Decision BGE 51 I 325).

##### The duty of the Federal State to protect and promote health

Respecting, protecting and promoting the health of the population living in Switzerland require different types of measures. Article 118 of the Swiss Constitution provides a list of areas in which the Federal State can adopt laws to protect its population’s health. It refers to the control of the use of food products, medical products, narcotics, organisms, chemical products and other objects that can present a health risk. The Federal State is also authorized to adopt measures to fight against communicable diseases, widespread disease and particularly dangerous diseases for human beings and animals. Finally, the article also lists the necessity for the Federal State to adopt measures to protect its population from ionizing radiation. In parallel, the Federal State also must take measures to ensure it meets the commitments it made in article 41 of the Federal Constitution. This article delineates the social goals the Federal State and the cantons aim to satisfy. They include the objective rights: for everyone to benefit from social security and necessary health care, to work, to shelter and to education. In this respect, article 117a provides that the Federal State, as well as the cantons, can take measures to ensure that everyone in Switzerland has access to sufficient basic medical treatment. Article 117 states that the Federal State can adopt laws in the field of the health insurance.

##### The right to get support in situation of distress

As a result of the primacy of the right to life and dignity enshrined in articles 10 and 7 of the Constitution, article 12 provides for the supply of essential subsistence means in situation of distress to all individuals in Switzerland. The existence of a duty for the State to provide support to indigent foreigners is not recent. In a 25th of September 1925 decision of the Federal Tribunal [25], the Court already confirmed this duty in the case of a Russian woman who had entered the country with her husband using counterfeit documents and who was seeking support from different cantonal authorities as she had no means to ensure her livelihood and that of her newborn son. While the husband had been arrested and incarcerated for fraud in the Cantons of Geneva, Vaud and Zurich, the issue before the Court was to decide which canton had to provide assistance to the woman and her son. In this decision, the duty to assist foreigners in situation of distress was seen as a duty of humanity and entrenched in the States responsibilities to ensure and protect public order. As we will see below in paragraph 2C, while this assistance is guaranteed to all, differences in the treatment of foreign nationals may exist in cantonal legislations. On the 27th of October 1995 [26], the Federal Tribunal went further and recognized the unwritten fundamental right to the minimum level of subsistence as it conditions the exercise of the right to life, to human dignity and the equality principle. The case concerned three Czech nationals—who had initially been admitted to Switzerland as refugees and then expelled for criminal offences and who subsequently re-entered the country illegally and could not be expelled again as the Czech Republic had rescinded their citizenship—and the Canton of Bern, which denied their right to social benefits. The Court held that the exclusion of three non-nationals from social welfare was a violation of an implied constitutional right to a basic minimum level of subsistence. In order to validate this decision, the Constitution was revised in 2000 to include what is now article 12. It provides for the delivery of the necessary essential means to live a life in conformity with the principle of dignity. This support includes basic health care, as well as other essential goods such as food, clothing and shelter.

#### The federal legislation to support the implementation of the right to health

##### The health insurance law

While article 12 of the Swiss Constitution ensures the provision of essential care to all people in situation of distress, and article 41 states that providing necessary health care to all is a social objective, the Federal State adopted the Federal Law of 18 March 1994 on Health Insurance (LAMal) in conformity with article 117 of the Federal Constitution. Considering that the provision of emergency health care in situation of distress is an exceptional situation, having access to the necessary health care is made possible in Switzerland through the obligation set by article 3 of LAMal for every person who lives in Switzerland for more than 3 months, to have his or her own health insurance. There is no distinction between residents: citizens, documented and undocumented foreigners, asylum seekers and refugees are obliged to subscribe to a health insurance. The corollary pillars of this obligation are the duty for health insurance companies to accept all requests for enrolment coming from individuals living in Switzerland irrespective of their residence status and the duty of the cantons to regulate residents and insurers ensuring they abide by their obligations. Considering the financial burden this obligation entails for residents, the 1994 LAMal provides the possibility to claim reductions in monthly premiums in the canton of residence. The amount of the reduction and the conditions to receive the benefit are fixed by each canton. While access to healthcare services is therefore, in principle, guaranteed to all, as everyone should have health insurance, we will see that the implementation of the obligation to have health insurance and the subsequent equal access of all to health care in Switzerland is subjected to practical problems in each canton.

##### The Asylum Act

Under article 121 of the Swiss Constitution, the authority for the granting of asylum is given to the Confederation. The Swiss Asylum Act of 26 June 1998 regulates the asylum procedures. The responsible authority for the application of the asylum law is the State Secretariat for Migration (SEM). While Switzerland is not a member of the European Union, it is nevertheless part of the EU-Dublin regulation [27], which establishes criteria and mechanisms for the responsibility for examining an application for international protection [28]. It provides that the State where an asylum claim is lodged is responsible for a person’s asylum claim. But at the same time, every State has the possibility to decide whether or not to examine an asylum claim [29]. Migrants who claim asylum at the border (Article 18 Asylum Act) or following an illegal entry in Switzerland are first transferred to a federal reception and procedure centre (Article 21 § 1 Asylum Act). A different procedure is in place to claim asylum at an airport (Article 22 Asylum Act). The Swiss Asylum Act does not foresee specific medical support at the arrival in the federal reception and procedure centres or in the airports’ centres. Care assistance is provided here by external organizations, and medical assistance is, for the most part, not offered by medical professionals [30]. Once the application for asylum has been completed in these federal reception and procedure centres, the preparatory phase begins (Article 26 § 1^quater^ Asylum Act). During this preparation phase and at the latest at the hearing on the grounds of asylum, asylum seekers are obliged to indicate any serious health problems of relevance to the asylum and removal procedures [31]. If asylum seekers claim medical problems later or if the medical problems are established by a medical specialist, they may be taken into account in the asylum and removal procedure if they are proven. If there are excusable grounds that an asylum seeker has not mentioned medical reasons with the deposit of the asylum claim, they could also be taken into account at a later time. In this case, the SEM can call in an independent medical examiner for verification of the medical problems [32]. After 90 days, asylum seekers are transferred to the reception and procedure centres in the cantons (Art. 16 § 2 Ordonnance 1 du 11 août 1999 sur l’asile relative à la procédure). At the cantonal level, the care assistance, including medical care, is also provided by external organizations [33].

After the establishment of the facts and after the hearing on the grounds of asylum, including personal details, travel and identity documents, itinerary and reasons for leaving their country, the SEM takes the decision on an asylum claim (Article 29 Asylum Act), based on criteria indicated in the Asylum Act (Article 31a). For those asylum seekers who have already claimed asylum in another country, the SEM takes the decision to remove the applicant to the other country or to treat the asylum claim itself after the Dublin State concerned has agreed to the transfer request (Article 37 § 1 Asylum Act). The SEM grants asylum to those who qualify for refugee status and if there are no grounds for denying asylum (Article 49 Asylum Act). Otherwise, the SEM rejects or dismisses the application for asylum and issues the removal order (Article 44 and 44a Asylum Act).

In cases in which the enforcement of removal or expulsion is not possible, not permitted or not reasonable, the SEM grants temporary admission (Article 83 § 1 of the Federal act of 16 December 2005 on Foreign Nationals (FNA)). Enforcement is not possible if the foreign national is unable to travel or cannot be brought back to their native country, to their country of origin or a third country (Article 83 § 2 FNA). Furthermore, enforcement is not permitted if Switzerland’s obligations under international law, like the principle of non-Refoulement (not forcing to return to a country where the asylum seeker’s life or his freedom would be threatened (Article 3 European Convention on Human Rights (ECHR))), prevent the foreign nationals from making an onward journey to their native country, to their country of origin or to a third country (Article 83 § 3 FNA). Finally, the enforcement may be unreasonable for foreign nationals if they are specifically endangered by situations such as war, civil war, general violence and medical emergency in their native country or country of origin (Article 83 § 4 FNA). Under certain medical circumstances, the execution of a removal order is not possible, not permitted or unreasonable, also in the context of article 3 ECHR. Article 3 ECHR postulates the principle of non-Refoulement, which provides that no one shall be subjected to torture or to inhuman or degrading treatment or punishment.

The European Court of Human Rights decided in D. v. the UK that an expulsion of an alien drug courier to St Kitts who was dying of AIDS violates article 3 ECHR. The Court concluded that there were no accommodation, family, moral or financial support and no access to adequate medical treatment for the person concerned. Therefore, in these very specific and exceptional circumstances, as recognized by the European Court of Human Rights in the case of D. v. the UK [34], the removal would violate article 3 ECHR. Almost 10 years later, the Court took a different decision. The asylum claim of a Ugandan woman, who was diagnosed with AIDS and was given high levels of immunosuppressive drugs, was rejected by the authorities of the UK. Subsequently, the UK decided to deport her back to Uganda. The woman claimed a violation of article 3 ECHR because of her illness and the lack of sufficient treatment available for it in her home country. In the case N. v. the UK [35], the Court states a non-violation of article 3 ECHR because there were no exceptional circumstances. The woman was not critically ill, like the applicant D., and even if her quality of life and her life expectancy would be affected, she could return to Uganda and obtain the medical treatment and support that she needs there.

In the case of Switzerland, the Federal Administrative Court also states in its jurisprudence that in certain specific health cases, an execution of the removal order is not possible, not permitted or unreasonable. In the case of AIDS-infected persons, the former Swiss Asylum Appeals Commission makes a distinction between persons who are in the final stage (AIDS) or at the beginning of their illness (HIV). The Swiss Asylum Appeals Commission adopted the jurisprudence of the European Court of Human Rights in a decision from October 2003. In this case, a Guinean was diagnosed with a late-stage AIDS infection and he was immediately medicated. The former Federal Migration Office for Refugees rejected his asylum claim and later also refused his demand for a temporary admission. They justified the decision based on the criminal activities of the Guinean man. The Swiss Asylum Appeals Commission also refused his complaint and explained that under certain circumstances, it could be a violation of article 3 ECHR if the authorities expulse a very ill person. In the present case, however, an expulsion did not violate article 3 ECHR because the Guinean had a good social network in his home country and his medical needs were guaranteed. Furthermore, since the Guinean had committed different crimes, a temporary admission, especially the question of not carrying out the removal order was unreasonable, could not be examined (JICRA 2004/6-037, 24.10.2003). A few months later, the Swiss Asylum Appeals Commission clarified and differentiated the jurisprudence in another judgement. The judges decided that a Cameroonian, infected with AIDS, could be expulsed. The question of the reasonability of the expulsion included not only the consideration of the phase of illness but also the consideration of the access to medical care in the home country (JICRA 2004/7-044, 13.01.2004). In September 2005, the Swiss Asylum Appeals Commission clarified the particular circumstances when an execution of a removal of a person with health problems violates article 3 ECHR. In the concrete case, a Bosnian woman and her children were confronted with an expulsion order. The children were diagnosed with psychological trauma because of their experiences during war and the woman was close to commit suicide (JICRA 2005/23-209, 14.9.2005). In the case Bensaid v. the UK [36], the judges considered that a removal order could violate article 3 ECHR if the access to health care was limited and the lack of treatment of the illness could bring self-harm. Therefore, a real risk, and not just a speculation of this risk, should be established. The Swiss Asylum Appeals Commission finally denied a real risk and therefore a violation of article 3 EHCR (JICRA 2005/23-209, 14.9.2005).

##### Federal legislation to protect the populations’ health through the promotion of the social determinants for health

In its scope of authority, the Federal State has adopted a number of laws that allow the effective protection and promotion of the right to health in Switzerland. In conformity with article 8 of the Swiss Constitution that enshrines the general principle of equality, this legislation benefits all residents in Switzerland. Take for example the case of the new Law on Epidemics (LEp) [37], which illustrates the intervention of the Federal State to ensure the protection of the population of its territory against communicable diseases. While article 41 of the new law provides for the possibility for people that enter Switzerland to undergo a medical check in the case of an outbreak of a communicable disease to limit its spread, the text does not differentiate amongst people entering, who can therefore be Swiss nationals, tourists or any other foreigners. Other laws may also be mentioned, for example the 2008 Federal Law on Passive Smoking of the 3rd of October 2008 that declared a prohibition of smoking in closed areas accessible to the public or that constitute a working environment for many individuals or the Federal Act on Research involving Human Beings adopted on the 30th of September 2011, which ensures the dignity, privacy and health of human beings involved in research.

Beyond these laws, which benefit all residents, Switzerland has also adopted measures aimed at integrating foreigners. The correlations between health and social integration are numerous, particularly as bad health is notably an impediment to education and work and may result in social exclusion [38]. Switzerland’s integration policy is based on the FNA and the Ordinance of 24 October 2007 on the Integration of Foreign Nationals (OIE), as well as all the cantonal laws related to the integration of foreigners. While the FNA focuses on foreign nationals with residence status, the fact that the LAMal requires that all people living in Switzerland for more than 3 months subscribe to a health insurance postulates that measures are being undertaken at the federal and cantonal levels to ensure that all foreign nationals can access and benefit from health services under the same conditions. One example of an impediment that may deter the effective use of the health system by foreign nationals is their incapacity to understand and to be understood. It undermines their ability to understand preventive measures [39], to follow doctor’s medical instructions and more generally to provide informed consent to the treatment they receive [40]. Some studies have proven that this lack of understanding leads to inadequate treatments [41]. Despite an increase in the number of requests for interpretation services in Swiss hospitals [42], there is no law ensuring the right to access to a medical interpreter in Switzerland and no legislation defining, organizing and financing interpretation services in hospitals for people who do not speak official Swiss languages. As public hospitals have an obligation under public law to provide health care to all people, interpretation will be sought if it is needed; however, this obligation is not enforceable for private practitioners such as family doctors in non-emergency situations [43]. Moreover, Ayer notes that in this circumstance, private practitioners can decide not to treat allophone patients [44]. The Administrative Federal Tribunal has rejected the possibility for such services to be financed through the basic health insurance, as interpretation is seen as a support measure, not a medical act [45]. Two parliamentary interventions that aimed at revising this law to include interpretation services were rejected [46]. Solutions must therefore be found at the cantonal level. In spite of this lack of legislation, the Federal Office for Public Health in the 2013 National Programme for Migration and Health emphasized the importance of interpretation services, dedicating an entire pillar of its 2014–2017 strategy to promotion of these services. The document highlighted the need to find innovative financing methods and development of training programmes in addition to providing access to community interpreters [47].

### The role of the cantons in the implementation of the right to health

Alongside the Federal State and within the field of health promotion and protection, the 26 cantons have relatively broad powers to ensure the implementation of the right to health in their territory. In this section, we analyse cantonal authority in the administration of emergency aid (A) and health insurance (B) in order to determine differences in treatment that may exist between foreign nationals and Swiss residents. We then outline how cantons ensure the provision of health care to asylum seekers (C) and the extent of their efforts to integrate allophone foreigners into the Swiss health system (D).

#### Cantonal authority on emergency aid

As there is no federal definition of what health services should be included in the emergency aid provision (article 12 of the Swiss Constitution), the notion is subject to different interpretations at the federal and cantons levels. While the Federal Tribunal referred to “basic medical care” in its 1995 decision, cantons have implemented different approaches in their respective legislations. For instance, the constitution of the Canton of Appenzell Ausserrhoden provides for “essential health care” [48]. Furthermore, in the Canton of Geneva, a December 2001 recommendation of the Advisory Board of Medical Ethics of the University Hospital of Geneva declared that all individuals should be entitled to receive all necessary vital medical care [49]. A specific unit was created in the University Hospital of Geneva, the Réseau Santé pour tous [50], to provide health care to socially vulnerable people and migrants. Additionally, the Consultation Ambulatoire Mobile de Soins Communautaires (CAMSCO) provides first recourse and general health care to vulnerable people and undocumented migrants. A similar entity, the Vulnerable Populations Unit, exists in the Lausanne University Medical Polyclinic (PMU). The two units have access to gatekeeping nurses and first recourse doctors, who provide access to other healthcare services if needed [51]. The units closely collaborate with non-governmental agencies and associations working with vulnerable populations. Multiple sources of financing exist to cover the costs for the treatment of these populations. One of these sources is the insurance company if the patient has enrolled, alternatively, the canton or the municipality using solidarity or social funds. In other cases, the healthcare institution can also finance these services. The patient may also be asked to pay. In other cantons, non-governmental organizations ensure healthcare services for undocumented migrants. In this respect, the Swiss Red Cross provides a wide scope of health services to vulnerable populations in the Cantons of Bern and Zurich—notably supplying health information and advice, basic health care, preventive care, psychiatric support and translation. Similarly, the Dispensaire des rues in the Canton of Neuchâtel employs nurses offering assistance to these populations within a dedicated healthcare network of doctors and dentists. The organization has existed for almost a hundred years and has developed strong links with the hospital of Neuchâtel.

#### Cantonal authority on the administration of health insurance

Cantons have the authority to grant reductions of monthly health insurance’s premiums (1) according to specific criteria. At the same time, they are charged to oversee (2) that all individuals residing in Switzerland for more than 3 months subscribe to health insurance and that insurance companies accept all requests for enrolment in basic health coverage.

##### Access to reduction of monthly health insurance premiums

According to a 2013 estimate of the Federal Office of Public Health, subscription to basic health insurance cost an average of 259 Swiss francs (CHF) per person per month [52]. In order to support individuals in a “modest economic situation,” the LAMal provides for possibilities to claim monthly premium reductions to the canton of residence (Article 65 § 1). The amount as well as the scope of the benefits and the conditions required to receive these reductions are fixed by each canton and can therefore vary according to the place of residence [53]. In order to determine the amount of the monthly reduction, the cantons calculate the core need income on the basis of taxable incomes and wealth. In this process, some cantons, such as the Canton of Aargau, require that the claimants provide an income tax statement to assess their needs. The consequence of this requirement is that individuals who do not pay taxes, and thus cannot provide an income tax statement, do not qualify for these reductions [54]. Despite small differences that exist amongst cantonal practices [55], all individuals who are likely to benefit from these reductions, are generally advised by the cantonal competent authority. Provided that they pay income tax, information and forms are sent directly to them. If no notification or documentation is sent, information is also available on official websites and sometimes through cantonal campaigns. The language barrier can here too be an additional impediment for migrants who do not speak national languages.

##### Duty of the cantons to enforce universal subscription to health insurance

Cantons are charged with the mission to inform the population living within their territories about the obligation to subscribe to health insurance (Article 10 of the Ordinance of 27 June 1995 on Health insurance, OAMal). They can enforce an immediate and automatic enrolment should they identify uninsured individuals (Article 6 LAMal). However, it is difficult for the cantonal surveillance authorities to fulfil this mission with regard to undocumented migrants who, by definition, are not known to cantonal authorities. In 2002, the Federal Office for Social Insurances reiterated the obligation of health insurers to accept all individuals living in Switzerland [56]. In case of breach of their obligations, insurers can be levied a fee of 5000 CHF maximum. In practice, it is very difficult to know whether or not undocumented migrants are actually insured, but it is believed that the vast majority do not have insurance [57] or only choose to enrol when they become seriously ill and expect to see high medical expenses [58]. This difference between theory and practice is mostly due to the fact that undocumented migrants are reluctant to come into contact with authorities or whatever represents state authority because of the fear of being identified and expelled to another country. This problem is not only true in concern to insurance coverage but also when it comes to actually going to the doctor or to the hospital.

#### The role of cantons in the provision of health care to asylum seekers

According to article 80 of the Asylum Act, the cantons must grant financial social aid for the daily needs and housing of asylum seekers awaiting decision that are assigned to them by the State Secretariat. The same principle applies for emergency aid under article 12 of the Swiss Constitution, which is granted to asylum seekers whose claim has been rejected. However, in the case of rejected asylum seekers, the Asylum Act stipulates a provision of non-cash support, hence in forms of payment in kind (Article 82 Asylum Act). The cantons also must ensure that their assigned asylum seekers can subscribe to health insurance. Article 82a of the Asylum Act authorizes the cantons to limit the asylum seekers’ choice to freely choose their health insurance, creating an exception to the principle set in the LAMal. In practice, the cantons select one health insurance company, normally with a low premium, and insure all asylum seekers with this company. The same article allows the cantons to limit—to a certain extent—the choice of health providers the asylum seekers have access to. On the financial side, the cantons can agree with the health insurance company to waive the insuree’s participation in costs (i.e. out of pocket expenses paid by the patient that are not normally covered by the health insurance). The Asylum Act also removes the right to premium reductions for asylum seekers and persons in need of protection without a residence permit and who are reliant solely or partly on social assistance (Article 82a § 7 Asylum Act).

#### The role of cantons in promoting migrants’ integration into the health system

Recommendations were made by a group of experts mandated by the Federal Office for public health in 2008, to find solutions to finance translation services in hospitals, retirement institutions, medical centres and home health services. This group of experts notably recommended cantons to introduce the “right to be understood” in all cantonal health legislation [59]. While no cantonal law has directly enshrined this right, certain cantonal norms provide direct and indirect legal basis for the use of interpreters. This is the case of the 1996 Law on integration and multicultural cohesion of the Canton of Neuchâtel, which provides for the possibility to have recourse to interpreters (Article 7 let. e of the Loi neuchâteloise du 26 Août 1996 sur l’intégration et la cohésion multiculturelle). This canton is known for its liberal migration policy, and this Law, which links integration to multicultural cohesion, is one of the first of its kind in Switzerland [60]. Furthermore, the Canton of Bern’s Law on the integration of the foreign population provides for the possibility for the canton and local authorities to sign service conventions to achieve certain integration measures (Article 21 of the Loi bernoise du 25 mars 2013 sur l’intégration de la population étrangère). While the canton did not sign any interpretation contract, some hospitals in the canton, such as the University Hospital of Bern, the University psychiatric clinic of Waldau and the cities of Bern and Biel, did [61]. In the absence of legislation in the field, the biggest of the five university hospitals in Switzerland, the University Hospitals of Geneva (HUG) has taken the lead in the area and has adopted regulations to support the development and use of professional interpreters for patients who do not speak national languages. Communication with patients is the second essential pillar of the HUG’ Charter for patients [62]. This principle provides for the delivery of all necessary information to patients, and in this respect, specific support should be provided to those who do not understand the national languages [63]. In parallel, the 2002 advice of the Clinical Ethics Committee of the HUG assured the right of all patients to benefit from the services of professional interpreters to ensure the communication of medical information. It states that recourse to lay internal interpreters should be kept for emergency situations and communication of non-medical information [64]. Since 1993, the HUG have worked in collaboration with the Geneva Red Cross, which coordinates the HUG with professional interpreters, to deliver interpretation services to patients who need it [65]. The HUG make an annual contribution of 80 000 CHF to the Geneva Red Cross for this work; however, this collaboration is not based on a service contract. The services are paid through the budget of the HUG, and it is free for patients. In parallel, the University Hospital of the Canton Vaud (CHUV) has developed a similar approach in favour of the recourse to interpreters for people who do not speak national languages. In the University Medical Polyclinic (PMU) of Lausanne, migrants’ visits account for 40 % of consultations. The Association Appartenances, which has the mission to promote the autonomy and quality of life of migrants, trains and hires community interpreters and organizes courses to raise the awareness of health staff on how to conduct a conversation with three persons, the patient, the healthcare provider and the interpreter. While the institutions often have easy access to internal bilingual staff to translate conversations with patients that do not speak national languages, this solution has limitations [66]. In practice, the institutions only have recourse to Appartenances’ interpreters in serious clinical or psychosocial situations [67] and the institutions pay directly for these services. In this context, there is a large gap that could be filled by initiatives at the cantonal and federal levels to increase the capacity of migrants who do not speak and understand Swiss languages to develop good communication with their doctors.

## Conclusions

As we have shown above, Switzerland is built on two tiers of legislation and implementation: the federal level and the cantonal level. The material presented here brings to light the advantages and disadvantages of this system, which can be improved upon in terms of both legislation and its implementation.

While, on the one hand, federalism allows for a greater adaptability of the law, it also leads to the coexistence of multiple cantonal systems that can apply different policies in the field. Notably, this phenomenon was shown in the example of the various existing conditions required to benefit from monthly premium reductions. The federal government sets up measures to offset these differences, in particular through the “migration and health” programme from the Federal Office of Public Health. The purpose of this programme is to identify the weaknesses within the system and to propose and implement measures aimed at fostering migrants’ access to health care. In other words, the federal government is aware of the current shortfalls of the system with regard to the legal and ethical aspects of the right to health of migrants.

In spite of these issues, proof of the good quality of the Swiss system was provided in the 2014 MIPEX-index [68] on access to health care for migrants, where Switzerland ranks second out of 38 countries, mostly from Europe and also from North America and Asia. This index analyses and classifies the countries’ legislation and draws recommendations for the countries with the weakest results. No recommendations were made for Switzerland, which is ranked second behind New Zealand. This does not, however, mean that the Swiss system is perfect—there is room for improvement.

While a number of factors, such as professional status, the existence of social support, lack of proficiency in national languages and often a history of violence in their country of origin, have been highlighted as possible explanations for the differences in the feeling of health of migrants highlighted in the introduction [69], it appears that the current Swiss legal framework for the implementation of the right to health of foreign nationals can be improved upon. Elements such as insufficient access to health services for asylum seekers coming from conflict areas should be better taken into account through laws to improve the current practice. In this context, the already recommended concept of the “right to be understood” should be re-opened. Due to the current influx of refugees, the question of the reliability of migrants’ access to health care and medical treatment becomes even more pressing. Being understood plays a crucial role in this context. Good practice, such as the example provided by the University Hospital of Geneva, should be actively promoted by the State.

Beyond the need to find solutions to overcome the effects of language barriers, future efforts should focus on assessing the health impact of the length of the asylum procedure and on increasing the ability of foreign nationals to learn how the health system works. In other words, efforts should be made to ensure foreign nationals have the capacity to control their life and health.

## Abbreviations

BGE: *Bundesgericht* (Swiss Federal Tribunal)
EFTA: European Free Trade Association
ECHR: European Convention of Human Rights
FNA: Federal Act on Foreign Nationals
LAMal: Federal Law on Health Insurance
SEM: State Secretariat for Migration
EU: European Union
